# Alpha-1 Acid Glycoprotein and Podocin mRNA as Novel Biomarkers for Early Glomerular Injury in Obese Children

**DOI:** 10.3390/jcm10184129

**Published:** 2021-09-13

**Authors:** Anna Medyńska, Joanna Chrzanowska, Katarzyna Kościelska-Kasprzak, Dorota Bartoszek, Marcelina Żabińska, Danuta Zwolińska

**Affiliations:** 1 Department of Pediatric Nephrology, Wroclaw Medical University, 50-367 Wrocław, Poland; danuta.zwolinska@umed.wroc.pl; 2 Department of Pediatric Endocrinology and Diabetology, Wroclaw Medical University, 50-367 Wrocław, Poland; joanna.chrzanowska@umed.wroc.pl; 3 Specialist Laboratory at the Department of Nephrology and Transplantation Medicine, Wroclaw Medical University, 50-367 Wrocław, Poland; katarzyna.koscielska-kasprzak@umed.wroc.pl (K.K.-K.); dorota.bartoszek@umed.wroc.pl (D.B.); marcelina.zabinska@umed.wroc.pl (M.Ż.)

**Keywords:** obesity, glomerular injury, alpha-1 acid glycoprotein, urinary mRNA expression of podocyte-associated proteins

## Abstract

Introduction: Obesity, which is a serious problem in children, has a negative impact on many organs, including kidneys, and obesity-related glomerulopathy (ORG) is an increasingly common cause of ESKD (end-stage kidney disease) in adults. Early-detected and -treated glomerular lesions are reversible, so it is important to find a useful marker of early damage. The study aimed to evaluate the albumin-to-creatinine ratio (ACR), urinary alpha-1-acid glycoprotein (α1-AGP), and mRNA of podocyte-specific proteins as indicators of glomerular injury and their relationship with the degree of obesity and metabolic disorders. Materials and Methods: A total of 125 obese children and 33 healthy peers were enrolled. Patients were divided into two groups, depending on SDS BMI values. ACR, α1-AGP, mRNA expression of nephrin, synaptopodin, podocin, and C2AP protein in urine sediment were measured. Results: ACR values did not differ between groups and were within the normal range. α1-AGP and mRNA expression were significantly higher in obese children compared with controls. mRNA expression of the remaining podocyte proteins was similar in both groups. No significant differences concerning all examined parameters were found depending on the degree of obesity. There was a positive significant correlation between α1-AGP and ACR. Conclusions: Increased α1-AGP before the onset of albuminuria suggests its usefulness as a biomarker of early glomerular damage in obese children. An increased podocin mRNA expression also indicates podocyte damage and may be linked to ORG development. The lack of increase in expression of other podocyte proteins suggests that podocin mRNA may be a more specific and sensitive biomarker. The degree of obesity has no impact on the tested parameters, but further studies are needed to confirm it.

## 1. Introduction

Over the past few decades, overweight and obesity have been recognized as one of the most serious public health problems worldwide, affecting not only adults but also children. It is estimated that in the United States obesity affects approximately one-fifth of the school-age population [1]. According to the World Obesity Forum, 20–35% of European children are overweight or obese [2]. Similar results were obtained by the Polish authors examining school-aged children [3]. Obesity and obesity-related metabolic diseases may lead to hypertension and diabetes, which are associated with an increased risk of chronic kidney disease (CKD).

The pathogenetic role of obesity in the development of adult CKD has been confirmed in 15–30% of patients, and a large meta-analysis showed that obesity is an independent risk factor for the development of CKD [4,5]. Moreover, it has been found that overweight and obesity are closely related to an increased risk of end-stage kidney disease (ESKD) [6]. Experimental and clinical studies have demonstrated that obesity promotes structural, hemodynamic, and metabolic alterations in the kidney, damaging different nephron segments, particularly the glomerulus. In 1974, an association was first reported between massive obesity and glomerulopathy, which is morphologically similar to focal segmental glomerulosclerosis (FSGS) [7]. Nowadays, obesity-related glomerulopathy (ORG) is an increasingly recognized cause of ESKD in the adult population.

Sodium reabsorption in the proximal tubules and in the loops of Henle plays a key pathogenic role in the development of ORG. Low sodium concentration in the macula densa dilatates afferent arterioles and leads to hyperfiltration, which increases renal plasma flow (RPF) and the glomerular filtration rate.

Abitbol et al., who studied a group of children with kidney disease and proteinuria, found that the glomerular volume was significantly greater in obese children than in the non-obese control [8]. It should be emphasized that reduced kidney function is for a long time asymptomatic and detected only incidentally. 

Given that obese children often remain obese in adulthood, and obesity-related complications, including the kidney, begin in childhood, efforts should be made to detect them as soon as possible. In the era of the obesity epidemic, this should be a special health challenge for pediatric nephrologists.

It has long been known that albuminuria, not detectable in standard urinalysis, is a useful early marker of glomerular damage, as it precedes the development of overt proteinuria. Recent studies indicate that alpha-1-acid glycoprotein (α1-AGP) is a better indicator of the permeability of the glomerular filtration barrier than albuminuria [9]. Alpha-1-acid glycoprotein is an acute-phase protein synthesized primarily in the liver. It has immunomodulatory properties and participates in pro- and anti-inflammatory activities by affecting the release or inhibition of cytokines (TNF-α, IL-1, IL-6) [10]. Experimental studies in mice have shown that increased α1-AGP levels help maintain metabolic balance by suppressing inflammation [11]. In humans, α1-AGP mRNA expression correlates with mRNA expression of both pro- and anti-inflammatory cytokines released by adipose tissue (TNF-α, IL-6, adiponectin) [12]. Authors suggest that α1-AGP attenuates the inflammatory process in human adipose tissue. However, El-Beblawy et al., who studied children and adolescents with type 1 diabetes, found that α1-AGP is an independent factor for diabetic microvascular complications and that its urinary concentration increases with overt nephropathy [13]. 

Podocytes are terminally differentiated and highly specialized cells of the visceral epithelium, which play a major role in maintaining the structure and function of the glomerular filtration barrier. A slit diaphragm, consisting of structural and signaling proteins, is a junction connecting foot processes of neighboring podocytes. In many glomerulopathies, injured podocytes detach from the glomerular basement membrane into the urine and cause filtration barrier leakage [14,15,16]. This process has been used to assess the activity and progression of glomerulonephritis in adults and children [15,16,17]. Alternatively, urinary mRNA expression of podocyte-associated proteins (synaptopodin, nephrin, podocin, podocalyxin, and CD2AP) can be assessed.

## 2. Objectives

The study aimed to evaluate the urinary excretion of albumin, α1-AGP, and mRNA of podocyte-specific proteins as indicators of glomerular injury in obese children and their association with the degree of obesity SDS BMI reference ranges and metabolic disorders.

## 3. Materials

We examined 125 children (68 girls) aged 8–17.9 years (mean 13.7 ± 2.84) treated for obesity in the outpatient clinic at the Department of Pediatric Endocrinology and Diabetology (Wroclaw Medical University, Wrocaw, Poland). We enrolled only children with simple obesity or without concomitant chronic or acute infectious diseases. We excluded patients with secondary obesity associated with endocrine disorders, genetic syndromes, or central nervous system diseases. The control group included 33 healthy peers (18 girls) aged 7.6–17.8 years (mean 12.9 ± 3) with normal body weight. Patients were divided into two groups, depending on their SDS BMI values. Patients’ characteristics are presented in Table 1. 

The study was conducted according to the guidelines of the Declaration of Helsinki, and approved by the Bioethics Committee of the Wroclaw Medical University (No. KB-376/2016). Informed consent was obtained from the parents and subjects above 16 years old. 

## 4. Methods

Anthropometric parameters in all enrolled patients were measured: height with Harpenden Stadiometer with 0.1 cm precision and weight with an electronic weight scale (SECA, Hamburg, Germany) with an accuracy of 0.05 kg. Body mass index was calculated according to the following formula: BMI = body weight (kg)/(height (m))^2^. According to the WHO recommendations, obesity was defined as 2 standard deviations (SD) above the reference median, and overweight as 1 SD above the reference median. BMI charts for Polish children population were used [3].

Serum and urine samples were tested to measure baseline serum lipid parameters (total cholesterol, HDL cholesterol, LDL cholesterol, and triglycerides), carbohydrate parameters (fasting glucose), renal function (creatinine), and hsCRP. Estimated creatinine clearance (eGFR), using the Schwartz formula [18], and insulin resistance index were calculated. In all patients, albuminuria, α1-AGP, and urinary creatinine levels and the glucose loading test were performed. Additionally, mRNA expression of podocyte-associated molecules (nephrin, synaptopodin, podocin, and C2AP protein) in urinary sediments were assessed, as markers for the presence of detachment podocyte in the urine.

Blood samples were drawn from cubital veins after an overnight fast, during routinely performed laboratory tests. Samples were clotted for 30 min, and centrifuged for 15 min at 3000 rpm. After separation, the serum was frozen at −80 °C until assayed. Serum creatinine, total cholesterol, HDL cholesterol, LDL cholesterol, glucose, insulin levels, and blood were assessed on the same day. 

Urine was collected from the first morning void (50–150 mL), which was then centrifuged at 800× *g* for 30 min at 4 °C. The supernatant was frozen at <−80 °C until assayed. The pellet was washed in 50 mL PBS and centrifuged at 800× *g* for 10 min at 4 °C. Then, the pellet was analyzed for mRNA expression of structural genes, and stabilized in RNA later fluid (24 h, 4 °C). Samples were stored at <−70 °C until assayed. Creatinine and α1-AGP were measured by ELISA kits supplied by R&D Systems, Inc. (Minneapolis, MN, USA). Urinary albumin was measured by ELISA kits supplied by Wuhan (EIAab Science Co., Ltd., Wuhan, China).

All assays were performed according to the manufacturers’ instructions. The concentration of parameters was expressed per milligram of creatinine in urine, except for albuminuria, which was expressed in milligrams per gram of urinary creatinine (mg/g).

## 5. Gene Expression

The research involved a real-time PCR analysis of urine sediment gene expression of NPHS1, NPHS2, CD2AP, and SYNPO genes, referenced to 18S rRNA or GAPDH. Total RNA was isolated from urine sediments with a QIAamp RNA Blood Mini Kit (Qiagen, Germany) according to the manufacturer protocol, including genomic DNA removal with RNase-free DNase (Qiagen, Germany). The samples were reversely transcribed with a High-Capacity RNA-to-cDNA Kit (Applied Biosystems, USA) according to the manufacturer protocol. The reaction mixes containing 0.9 µL of reverse transcription product in 10 μL of Taqman Fast Advanced Master Mix were applied to each well of custom-designed plates (Taqman), including the following assays in triplicate: Hs00190446_m1 (NPHS1), Hs00387817_m1 (NPHS2), Hs00961458_m1 (CD2AP), Hs00200768_m1 (SYNPO), Hs99999905_m1 (GAPDH), and Hs99999901_s1 (18S rRNA). Amplification reaction was performed on Taqman 7900 HT instrument with a fast protocol and analyzed with SDS 2.2.2 software. The results are presented as ΔCT = CT_target_ − CT_reference_, or as ΔΔCT = ΔCT_mean control sample_ − ΔCT_test sample_. Mean control sample was the mean value of ΔCTs in the healthy control group.

## 6. Statistical Analyses

The results are expressed as mean (x), median (M), range (min–max), lower and upper quartiles (25–75 Q), and standard deviations (SD). The equality of means in independent groups was tested with ANOVA analysis of variance and in heterogeneous groups with a Mann-Whitney non-parametric U-test; homogeneity of variance was assessed by Bartlett’s test. For discrete parameters, we analyzed the frequency of trait occurrence using the c2df test with Yates’ correction with an appropriate number of degrees of freedom df (df = (m − 1) * (*n* − 1), where m—the number of rows, *n*—the number of columns). For correlation analysis of selected variables, we used the Pearson or Spearman’s correlation coefficient. A value of *p* ≤ 0.05 was considered statistically significant. Statistical analysis was performed with EPIINFO v. 7.1.1.14 statistical software package.

## 7. Results

The parameters of lipid metabolism differed significantly between the study group and the control group in terms of triglycerides and HDL cholesterol values. Total cholesterol and LDL cholesterol levels in both groups were comparable. Estimated creatinine clearance values also differed between the groups and were significantly higher in the obese child population. Fasting glucose was above normal in only four children in the study group. Detailed data are presented in Table 2.

Albumin excretion rate did not differ between the study and control groups, with values within normal range in all children (<30 mg/g creatinine). The excretion of α1-AGP was significantly higher in obese children compared with the healthy control. Detailed results are presented in Table 3. 

However, no differences were shown between the degree of obesity and albumin, and α-1-AGP levels (Table 4). 

The mRNA expression of nephrin, synaptopodin, and CD2AP in urine sediment was not significantly different between the groups, irrespective of the degree of obesity. Podocin mRNA expression was significantly higher in the group of obese patients compared with the non-obese group. Unexpectedly, it was significantly higher in children with a lower degree of obesity than in those with a higher degree of obesity. The results are shown in Table 5.

We found a positive correlation only between α1-AGP and albuminuria (*r* = 0.95; *p* = 0.000), and between α1-AGP and hsCRP (*r* = 0.54; *p* = 0.000).

## 8. Discussion

The study showed for the first time significantly increased α1-AGP excretion in the urine of obese children as compared with non-obese peers in the absence of overt albuminuria, considered so far as an early marker of glomerular damage. Burgert et al., among others, reported microalbuminuria in more than 10% of obese American children without other comorbidities [19]. According to the authors, the appearance of albuminuria depends primarily on hyperglycaemia, which was found in our study only in four children, which may have had an impact on the ACR result.

In the study by Lurbe et al., the prevalence of microalbuminuria in obese children was only 2.4%. These authors showed no relationship between the degree of obesity and increased albumin-to-creatinine ratio (ACR), which is consistent with our observations [20]. Sawamura et al. observed albuminuria in 21% of 64 overweight and obese children and adolescents, which did not correlate with eGFR, indicating that hyperfiltration is not the only factor that damages the glomerular filtration barrier in obesity [21].

Recent studies indicate that α1-AGP is a more sensitive marker of glomerular filtration barrier injury than albuminuria. This was proved, among others, by Talks et al., who studied the urinary α1-AGP and albumin leakage in healthy volunteers in an altitude-induced hypoxia (5023 m). These authors reported a significant increase in urinary leakage of albumin and α1-AGP, but the percentage of increase was significantly higher for α1-AGP. They observed no increase for positively charged dimeric lambda free light chains (λ-FLCs) [9]. It should be emphasized that the molecule of α1-AGP is smaller than the molecule of albumin (43 kDA vs. 66 kDA) and similar in size to the podocyte slit diaphragm pore. Similar observations were reported in adult patients with type 2 diabetes. Both Christiansen et al. [22] and Narita et al. [23] have shown increased urinary α1-AGP excretion in diabetic patients with normoalbuminuria. Similarly, in a study of 60 children with type 1 diabetes, α1-AGP excretion preceded the onset of microalbuminuria and significantly increased with its onset [13]. Moreover, these authors reported a significant positive correlation between α1-AGP excretion and albuminuria, which is in line with our observations. Therefore, it seems that urinary α1-AGP may be a useful marker for assessing glomerular injury in obese children. However, in our study we did not show any significant differences in urinary α1-AGP according to the degree of obesity. 

The mechanism of glomerular injury in obese patients is complex and not fully understood. Certainly, hemodynamic and metabolic as well as endocrine disturbances are interconnected. For this reason, the effect of damage to individual kidney structures may not be the same in all obese patients. Although, according to Tsuboi et al., severe forms of ORG affect mainly morbidly obese patients, the severity of kidney damage is not necessarily related to the severity of obesity [24].

Alpha-1-acid glycoprotein, a major acute-phase protein, displays a number of anti-oxidative and anti-inflammatory properties [25]. For this reason, its increased levels in serum and urine have been linked to vascular inflammation and subclinical atherosclerosis. This has been confirmed by El-Beblawy et al., who found a significant correlation of serum and urinary α1-AGP with hsCRP and cartoid intima-media thickness (IMT) in children with type 1 diabetes [13]. 

It is very likely that in obese children, α1-AGP may be a good marker not only of glomerular injury but also of very early atherosclerotic lesions. In this study, we found also a significant positive correlation between the α1-AGP and hsCRP. 

Yang et al. have shown that in patients with cardiovascular disease, elevated levels of high-sensitivity CRP increase the risk of developing microalbuminuria [26]. It cannot be excluded that we would observe a similar association with longer duration of obesity. In fact, we found significantly higher hsCRP concentrations in obese children compared with non-obese children.

Many studies have demonstrated an association of podocyturia and mRNA expression of podocyte proteins in urinary sediment with an activity and progression of different glomerulopathies, including adult diabetic nephropathy [27] and lupus nephropathy [28].

However, only a few studies have demonstrated this association with ORG and the “silent” glomerular damage in obesity. For instance, Chen et al. reported a significantly lower number of podocyte density in 46 adults with clinically overt ORG compared with healthy controls [29]. Moreover, these changes were strongly associated with proteinuria and abnormalities in fasting glucose and insulin levels [29]. However, Pereira et al., who studied urinary expression of podocyte-associated mRNAs, showed higher expression levels of podocin and nephrin mRNAs in obese and overweight adults as compared with healthy controls. Moreover, it was associated with the severity of obesity and metabolic syndrome. What is, however, worth emphasizing is that they did not detect albuminuria [30]. 

However, higher serum insulin levels were related to podocyturia, regardless of obesity. Our study, which is, to our knowledge, the first one conducted in a population of obese children, is consistent with the previous results only with regard to podocin mRNA, because the mRNA expression of other podocyte proteins did not differ between the obese children and the healthy control. Moreover, our study did not confirm the association of podocin mRNA with weight gain or metabolic disorders, which may be due to the fact that our population was younger as compared with the obese population in other studies. The above results confirm that glomerular lesions assessed by mRNA expression of podocyte proteins occur even before the onset of albuminuria. The significantly higher mRNA expression levels of podocin, and not of other proteins, are difficult to explain. Some suggest that podocin may be a more specific and sensitive marker in podocyte damage than nephrin, podocalyxin, or synaptopodin. This was confirmed, among others, by Garovic et al., who conducted investigations in pregnant women with preeclampsia [31]. This is also suggested by the results of experimental studies on rats with diphtheria-induced kidney damage with a subsequent increase in urine podocin and nephrin mRNA expression [32]. After 8 days of observation, the presence of podocin mRNA was still detected, and no expression of nephrin mRNA was found. Given the fact that obese patients may develop ORG, podocyturia measured with urinary mRNA expression levels of podocyte-assosiated proteins may be one of the first indicators of ORG development.

## 9. Conclusions

Increased α1-AGP before the onset of albuminuria suggests its usefulness as a biomarker of early glomerular damage in obese children. An increased podocin mRNA expression also indicates podocyte damage and may be linked to ORG development. The lack of increase in expression of other podocyte proteins suggests that podocin mRNA may be a more specific and sensitive biomarker. The degree of obesity has no impact on the tested parameters, but further studies are needed to confirm it.

## Figures and Tables

**Table 1:** Patients’ characteristics according to the SDS BMI values.

	Subgroup I 2 ≤ SDS BMI ≤ 4	Subgroup II SDS BMI > 4	*p*
Number of patients	65	60	
Age (years) (mean ± SD)	13.7 ± 2.8	13.5 ± 2.9	NS
range	8–17.9	8.2–17.8	
Sex (F/M)	29/31	29/21	NS
(%)	48.3/51.7	58/42	

NS—insignificant statistical difference, SD—standard deviation.

**Table 2:** Biochemical parameters in study and control group.

		Control Group F/M 18/15	Study Group F/M 68/57	*p*
Total cholesterol (mg/dL)	mean ± SD	164.1 ± 14.8	179.2 ± 134.9	0.537
	range (min–max)	133–188	111–1611	
	median	165	166	
	quartile (25–75 Q)	155–175	145–187.5	
HDL cholesterol (mg/dL)	mean ± SD	59 ± 9.6	42.2 ± 8.4	0.00000
	range (min–max)	34–78	27–65	
	median	62	42	
	quartile (25–75 Q)	50–67	36–48	
LDL-cholesterol (mg/dL)	mean ± SD	94.1 ± 14.9	100.4 ± 24.4	0.175
	range (min–max)	65–121	49–184	
	median	97	99	
	quartile (25–75 Q)	81–105	82–118	
Triglycerides (mg/dL)	mean ± SD	90.3 ± 17.8	122.5 ± 62.1	0.00216 *
	range (min–max)	57–120	39–469	
	median	94	107	
	quartile (25–75 Q)	74–105	83.5–141	
Creatinine (mg/dL)	mean ± SD	0.736 ± 0.144	0.629 ± 0.123	0.00004
	range (min–max)	0.54–1.19	0.37–0.89	
	median	0.69	0.615	
	quartile (25–75 Q)	0.65–0.81	0.54–0.71	
eGFR (mL/min/1.73 m^2^)	mean ± SD	125 ± 13.2	154 ± 25.1	0.00000
	range (min–max)	96.0–160.0	109–235	
	median	123.5	152	
	quartile (25–75 Q)	117.5–132	137–169	
Fasting glucose (mg/dL)	mean ± SD		82.5 ± 10.4	0.0118 *
	range (min–max)	75–94	56–153	
	median	88	82	
	quartile (25–75 Q)	85–91	77–82	

For a distribution deviating from the normal distribution, no mean was calculated. * analysis with the non-parametric Mann-Whitney U test.

**Table 3:** ACR, α-1AGP, and hsCRP in the study and control groups.

		Control Group	Study Group	*p*
		*n* = 33	*n* = 125	
ACR (mg/g) albumin-to-creatinine ratio	mean ± SD	14.53 ± 1.11	13.71 ± 2.75	0.168 *
	range (min–max)	12.86–16.02	12.78–18.03	
	median	15.17	14.7	
	quartile (25–75 Q)	13.42–15.51	12–15.64	
α-1 acid glycoprotein (ng/mg)	mean ± SD	447.9 ± 37.9	578.5 ± 165.2	0.00000 *
	range (min–max)	378.5–500	126.3–1783	
	median	463.3	631.4	
	quartile (25–75 Q)	407.7–479.2	470.1–672.6	
Serum hsCRP (µg/mL)	mean ± SD	1.22 ± 0.11	3.24 ± 0.44	0.00000 *
	range (min–max)	1.02–1.45	2.36–4.46	
	median	1.2	3.22	
	quartile (25–75 Q)	1.14–1.32	2.86–3.54	

* analysis with the non-parametric Mann-Whitney U test.

**Table 4:** Glomerular damage indicators between groups of children depending on the degree of obesity.

		2 ≤ SDS BMI ≤ 4	SDS BMI > 4	*p*
		*n* = 60	*n* = 50	
ACR (mg/g)	mean ± SD	13.6 ± 2.6	13.7 ± 3.2	0.945
	range (min–max)	1.89–6.4	1.8–18	
	median	14.6	15.1	
	quartile (25–75 Q)	12–15.7	12–15.6	
α-1 acid glycoprotein (ng/mg)	mean ± SD	590.7 ± 197.2	564 ± 137.6	0.421
	range (min–max)	131–1783	126.3–727.8	
	median	631.8	633.3	
	quartile (25–75 Q)	469.4–674.1	472.8–679.9	
Serum hsCRP (µg/mL)	mean ± SD	3.17 ± 0.41	3.31 ± 0.45	0.103
	range (min–max)	2.36–4.08	2.55–4.64	
	median	3.19	3.29	
	quartile (25–75 Q)	2.84–3.46	3.05–3.59	

Analysis with the non-parametric Mann-Whitney U test.

**Table 5:** The level of mRNA expression of the podocin gene between groups of children depending on the degree of obesity, expressed as ΔΔCt.

Gene Examined	Gene Reference	Examined Value	2 ≤ SDS BMI ≤ 4 *n* = 17	SDS BMI > 4 *n* = 16	*p*
NPHS2	18S	range (min–max)	0.51–10.24	(−1.01)–5.48	0.017 *
		median	2.21	1.2	
		quartile (25–75 Q)	1.68–7.5	0.00–2.12	
NPHS2	GAPDH	range (min–max)	1.13–8.44	(−2.03)–7.13	0.001 *
		median	2.56	0.085	
		quartile (25–75 Q)	1.83–5.18	(−0.005)–1.815	

For a distribution deviating from the normal distribution, no mean was calculated. * analysis with the non-parametric Mann-Whitney U test.

## Data Availability

Data associated with the paper are not publicly available but are available from the corresponding author at reasonable request.
